# Switching focus enhances swimming performance and attentional flexibility in beginner swimmers

**DOI:** 10.3389/fpsyg.2026.1853379

**Published:** 2026-07-13

**Authors:** KaiJun Wang, Dan Li, Liang Zhao, Can Feng, Chi-Lun Tsai

**Affiliations:** 1 Ice and Snow College, Jilin Sport University, Changchun, China; 2 College of Leisure and Social Sports, Jilin Sport University, Changchun, China; 3 Graduate Department, Jilin Sport University, Changchun, China; 4 Department of Sport Psychology, Leipzig University, Leipzig, Germany

**Keywords:** attentional flexibility, attentional focus, child athletes, performance, swimming

## Abstract

Previous studies have shown that directing attention externally can facilitate more fluent and automatic motor control, whereas an internal focus of attention may introduce excessive conscious regulation and impair performance in land-based tasks such as archery, shooting, and golf putting. However, emerging evidence suggests that switching attentional focus may be functionally beneficial for performance in complex motor skills that require continuous coordination of multiple body segments, such as swimming. However, prior research has rarely manipulated switching attentional focus, leaving a critical gap in understanding how dynamic attentional regulation influences motor performance. To address this gap, the present study examined whether a switching focus strategy would lead to superior swimming performance compared to fixed internal or external focus strategies. A total of 20 novice male swimmers (*M_*age = 11.50 years, *SD* = 0.76) performed 25-m front-crawl sprints under four conditions: no-instruction control, internal focus, external focus, and switching focus. The results showed a significant condition effect on swimming time, *F*(3, 57) = 16.20, *p* < 0.001, ηp^2^ = 0.460, with the switching-focus condition producing shorter swimming times than both the internal-focus and external-focus conditions; however, no significant difference was observed between the internal-focus and external-focus conditions. In addition, a significant condition effect was found for attentional flexibility, *F*(3, 57) = 26.10, *p* < 0.001, ηp^2^ = 0.579, with the switching-focus condition showing higher attentional flexibility than the control, internal-focus, and external-focus conditions. Both internal-focus and external-focus conditions also demonstrated higher attentional flexibility than the control condition. However, no significant difference in attentional flexibility was observed between the internal- and external-focus conditions. These findings suggest that adopting a switching attentional focus may enhance task-specific perceived attentional flexibility and is associated with improved performance in complex motor skills, highlighting the importance of dynamic attentional regulation in swimming.

## 1 Introduction

Accumulating evidence shows that attentional focus instructions can meaningfully influence motor performance (Nicklas et al., 2022; Wang et al., 2021; Wulf et al., 2001; Wulf, 2013). In particular, attentional focus refers to the specific target to which performers direct their mental resources during action, commonly categorized as external focus (directed toward the effects of the movement) and internal focus (directed toward the body or movement mechanics; Wulf et al., 2001; Wulf and Shea, 2002). Previous studies have shown that directing attention externally can facilitate more fluent and automatic motor control, whereas internal focus may introduce excessive conscious regulation and impair performance (Nicklas et al., 2022; Wulf and Lewthwaite, 2016). To explain this phenomenon, the constrained-action hypothesis suggests that directing attention to one’s own body during movement can disrupt the automatic processes that normally guide motor control. In contrast, focusing on the effect of the movement in the environment allows these automatic processes to function more efficiently, leading to better performance and improved learning (Wulf et al., 2001; Wulf and Shea, 2002). However, this view may not fully apply to all skills. In complex tasks that rely on the moment-to-moment coordination of many body segments, an internal focus may not disrupt performance and may even be necessary to maintain proper control.

Swimming is one such case, as effective performance depends on continuous multi-segment coordination under limited sensory feedback (Messinis et al., 2014). Unlike land-based tasks, such as archery, shooting, and racing, swimmers cannot rely on stable visual cues and must instead integrate tactile and kinesthetic sensations to maintain stroke rhythm, breathing timing, and propulsion efficiency. Small disruptions in attentional control can quickly affect stroke mechanics, hydrodynamics, and pacing, ultimately compromising speed. Previous studies involving trained swimmers have demonstrated that internal focus cues can slow swim times, whereas external focus either maintains or enhances performance (Saemi et al., 2023; Freudenheim et al., 2010).

Interestingly, more evidence suggests that switching attentional focus may be functionally beneficial for motor performance. For example, in qualitative studies, Oliver et al. (2020) observed that athletes switched their attentional focus between external and internal foci during complex motor skills. Similarly, Bahmani et al. (2019) found that athletes who switched both foci of attention in a difficult situation showed superior skill execution. Furthermore, Wang et al. (2021), who adopted a psychophysiological approach, observed that athletes tend to switch their attentional focus (i.e., from external focus of attention to internal focus of attention) to have superior motor performance. In the present study, attentional flexibility refers to the perceived ability to adjust or redirect attentional focus during task execution in response to ongoing movement demands. This construct is particularly relevant to swimming because swimmers must continuously coordinate arm actions, leg movements, breathing, body position, and propulsion while receiving limited visual feedback. From this perspective, switching attentional focus may support performance by allowing swimmers to flexibly alternate between movement-related cues and task-level effects, rather than maintaining a single attentional focus throughout the task. However, previous research had neither directly assessed subjective attentional flexibility nor systematically manipulated switching attentional focus strategies. Thus, it remains unclear whether switching attentional focus enhances performance through increased attentional flexibility, leaving a critical gap in our understanding of how dynamic attentional regulation influences motor performance (Saemi et al., 2023; Freudenheim et al., 2010).

To test this assumption, the present study examined whether a switching focus strategy would lead to superior swimming performance compared with a fixed internal or external focus (Hypothesis 1), as it allows greater flexibility in attentional allocation (Hypothesis 2) during swimming. Furthermore, beginner swimmers were recruited because they are more sensitive to attentional manipulations, making them an appropriate population for detecting performance differences and underlying attentional mechanisms.

## 2 Materials and methods

### 2.1 Participants

The present study included 20 male swimmers (Table 1). The study was designed with one within-subject factor (different attention conditions). We used a sensitivity analysis in G*Power to indicate that with *N* = 20, the minimum detectable effect size for the within–between interaction was *f* = 0.45 at 80% power and *α* = 0.05 (Faul et al., 2007). Participants were novice swimmers with less than 1 year of front-crawl swimming experience who were able to perform the front crawl. The inclusion criterion was the absence of any diseases or musculoskeletal injuries. All procedures conformed to the ethical standards outlined in the Declaration of Helsinki and were approved by the Research Ethics Committee of Jilin Sport University.

**Table 1:** Descriptive statistics for participant characteristics by group.

Characteristic	*N* = 20	Mean	SD
Age (years)	Beginner	11.50	0.76
Height (cm)	Beginner	154.50	5.78
Weight (kg)	Beginner	34.60	2.95
Experience (years)	Beginner	0.620	0.2

SD, standard deviation.

### 2.2 Measures

#### 2.2.1 Visual analog scale for anxiety (VAS)

To ensure that anxiety is not a potential confounding factor, the VAS was used to assess participants’ anxiety levels in each condition. These assessments utilized a scale ranging from 0 (no anxiety) to 11 (extreme anxiety; Wang et al., 2023).

#### 2.2.2 VAS for attentional flexibility

To assess attentional flexibility, participants rated their perceived ability to flexibly allocate their attentional focus after completing swimming under each condition, using a scale ranging from 0 to 11 ranging from 0 (no perceived attentional flexibility) to 11 (maximum perceived attentional flexibility). The use of this single-item VAS was guided by previous studies that suggest dynamic shifts in attentional focus may be relevant to superior motor performance (Wang et al., 2021). However, because this VAS has not been validated as a standardized measure, the scores were interpreted as task-specific perceived attentional flexibility.

#### 2.2.3 Performance measurement

An electronic stopwatch was used to measure their performance (± 0.01 s accuracy). Timing began when the participant pushed off the pool wall at the start of each trial and stopped when the swimmer’s hand made clear contact with the opposite wall.

### 2.3 Task and procedure

The research design was similar to that of Saemi et al. (2023). First, participants were informed that the study examined swimming performance. They were then given a short, standardized presentation that introduced the concept of attentional focus and explained how the intervention would be implemented, without providing specific cue details to minimize potential confounding effects. Prior to participation, informed consent was obtained from participants’ parents.

Subsequently, each participant completed a 10-min in-water warm-up consisting of light front-crawl swimming. The experimental task involved repeatedly performing 25-m front-crawl trials, consistent with protocols used in prior attentional-focus research in swimming (Freudenheim et al., 2010; Stoate and Wulf, 2011). Participants were instructed to swim as fast as possible in every trial. Following the warm-up, beginners performed a baseline 25-m trial without receiving any attentional focus instruction (no-focus condition).

After the baseline trial, participants were introduced to the attentional focus conditions. The attentional cues were developed in consultation with experienced swimming coaches to ensure ecological validity and alignment with common coaching practices. Importantly, the internal-focus and external-focus cues were matched in movement content, differing only in the direction of attention.

To ensure consistent delivery across participants, the instruction followed a standardized sequence: (a) explanation of the attentional focus concept, (b) introduction of the specific cue, (c) guided rehearsal during the rest interval, and (d) a reminder immediately before each trial. This structured instructional sequence was intended to make the attentional-focus manipulation more explicit and consistently applied, in line with previous sport psychology intervention studies that used structured cueing, rehearsal, reminders, or imagery-based procedures to support athletes’ implementation of psychological strategies (Hatzigeorgiadis et al., 2004, 2014; Devonport et al., 2016).

The specific attentional focus instructions were as follows:

- Internal-focus condition: Participants were asked to direct attention toward their own movement mechanics, specifically emphasizing pushing their hands down and backward and whipping their feet through the water.
- External-focus condition: They were reminded to concentrate on moving the water down and behind them. These cues were adapted from earlier swimming studies.
- Switching-focus condition: Participants were asked to concentrate on moving the water down and behind them during the first half of the 25-m trial and then switch their attention to pushing their hands down and backward during the second half of the trial. Thus, the switch occurred at approximately the midpoint of the 25-m trial and followed a predefined external-to-internal focus order instructed by the experimenter before each trial, with no additional experimenter cue provided during the swim, rather than being freely selected by participants. This external-to-internal sequence was informed by previous psychophysiological evidence suggesting that performers may shift from an external to an internal focus during superior motor performance (Wang et al., 2021).

A rest interval of approximately 5 min separated each trial. During the rest interval, participants were reminded of the assigned cue and guided by the experimenter to ensure understanding and readiness. Before each trial, participants were again reminded of the specific attentional focus instruction and asked to verbally confirm their understanding.

Participants completed three trials under each of the three attentional focus conditions (external, internal, and switching), with condition order counterbalanced across participants. To minimize learning effects, no performance feedback (knowledge of results) was provided throughout the experiment.

**Figure 1:**
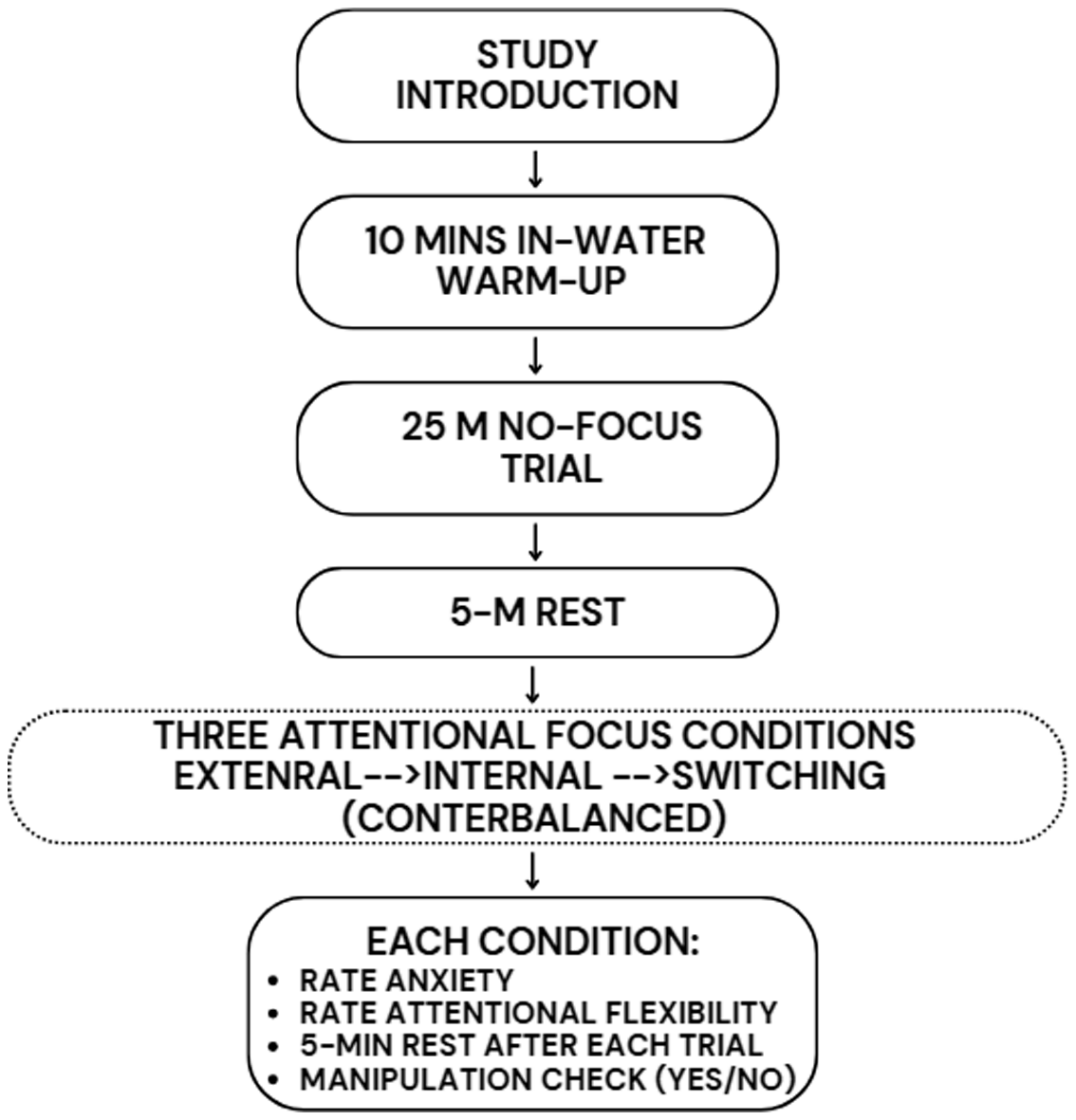
Overview of the experimental procedure.

At the end of each attentional focus condition, a brief manipulation check was conducted. Participants were asked to indicate whether they had followed the instructed attentional focus (yes/no) and to briefly describe what they had focused on during the trials. Finally, upon completion of the experiment, participants were asked not to discuss the experimental procedures with other participants (Figure 1).

### 2.4 Statistical analysis

Statistical analysis was performed using SPSS version 26.0 (IBM). A one-way repeated-measures ANOVA with condition (control, internal-focus, external-focus, and switching-focus) as the within-subject factor was performed on anxiety scores to examine whether anxiety differed across conditions. In addition, a one-way repeated-measures ANOVA on swimming performance was used to test Hypothesis 1, and a one-way repeated-measures ANOVA on VAS score (attentional flexibility) was used to test Hypothesis 2.

When significant effects of conditions were found, *post-hoc* analyses were performed using Bonferroni-adjusted pairwise comparisons to control for type I error inflation from multiple comparisons. The alpha level for all statistical tests was set at 0.05.

## 3 Results

### 3.1 Manipulation check

A brief self-report manipulation check was conducted to examine whether participants adhered to the instructed attentional focus strategy. The results indicated complete adherence across all attentional-focus conditions. Specifically, all participants reported that they followed the instructions in the internal-focus (yes = 100%, no = 0%), external-focus (yes = 100%, no = 0%), and switching-focus conditions (yes = 100%, no = 0%). However, this check was not designed to quantify the exact frequency, timing, or consistency of attentional switching during swimming.

### 3.2 Anxiety level

To control for a potential factor, a one-way repeated-measures ANOVA revealed no significant condition effect, *F*(3, 57) = 0.445, *p* = 0.722, *η_p_^2^* = 0.023.

### 3.3 Attentional flexibility (hypothesis 2)

**Figure 2:**
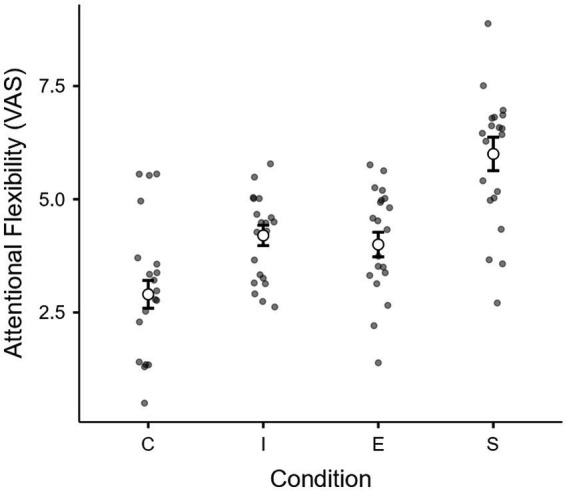
Estimated marginal means of attentional flexibility (VAS) across conditions. Error bars represent ±1 standard error of the mean. Individual data points are shown as gray dots. C, control condition; I, internal-focus condition; E, external-focus condition; and S, switching-focus condition. Higher VAS scores indicate greater perceived flexibility in attentional allocation.

A one-way repeated-measures ANOVA revealed a significant condition effect, *F*(3, 57) = 26.1, *p* < 0.001, *η_p_^2^* = 0.579 (Figure 2). A *Post-hoc* analysis indicated that attentional flexibility in the switching condition was significantly higher than in the control, internal-focus, and external-focus conditions (all *p* < 0.001); however, no significant difference was observed between the internal-focus and external-focus conditions (*p* = 0.33). In addition, both internal-focus and external-focus conditions showed significantly higher attentional flexibility than the control condition (*ps* ≤ 0.046).

### 3.4 Swimming performance (hypothesis 1)

**Figure 3:**
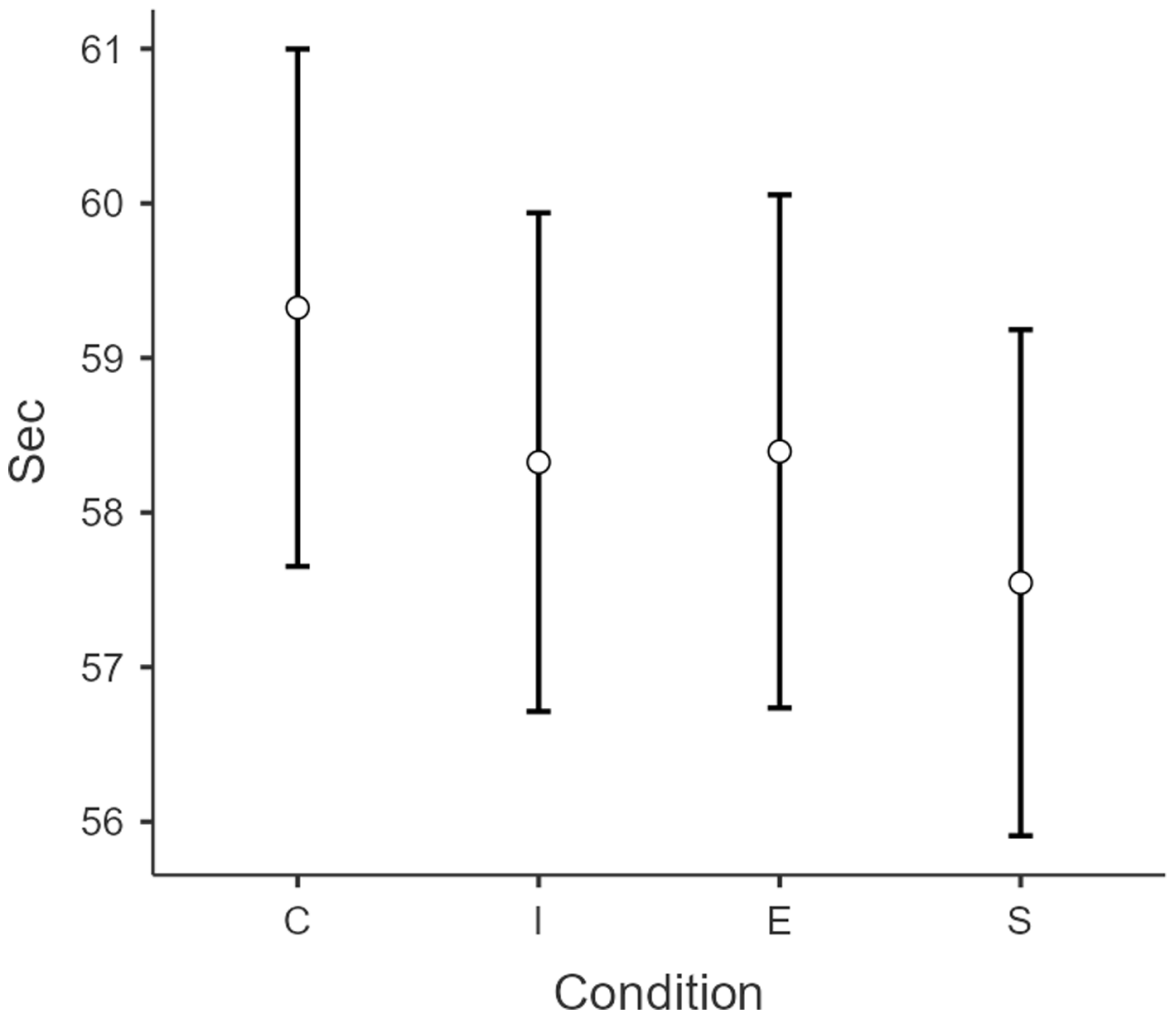
Mean swimming completion times (in seconds) across conditions for beginner swimmers. Points represent condition means with corresponding error bars. Gray dots represent individual participant data. C, control; I, internal-focus; E, external-focus; and S, switching-focus.

A one-way repeated-measures ANOVA revealed a significant condition effect, *F*(3, 57) = 16.2, *p* < 0.001, *η_p_^2^* = 0.460 (Table 2; Figure 3). A *Post-hoc* analysis using Bonferroni correction indicated that swimming times in the control condition were significantly longer than those in the internal-focus, external-focus, and switching conditions (all *ps* ≤ 0.019). In addition, both the internal-focus and external-focus conditions resulted in significantly longer swimming times compared to the switching condition (*ps* ≤ 0.04). In contrast, no significant difference was observed between the internal-focus and external-focus conditions (*p* = 0.99).

**Table 2:** Descriptive statistics for task completion time (sec).

Condition	Mean (s)	SE	95% confidence interval	
			Lower	Upper
Control	59.3	1.67	55.8	62.8
Internal-focus	58.3	1.61	55.0	61.7
External-focus	58.4	1.66	54.9	61.9
Switching-focus	57.5	1.64	54.1	61.0

## 4 Discussion

The purpose of the present study was to examine whether a switching focus strategy would result in superior swimming performance compared to fixed internal or external focus strategies (Hypothesis 1) and whether such a performance advantage could be explained by greater flexibility in attentional allocation during swimming (Hypothesis 2). To the best of our knowledge, this is the first study to directly examine the effects of a switching focus strategy on swimming performance and attentional flexibility in beginner swimmers. The results support both hypotheses. Specifically, swimmers achieved faster completion times in the switching condition than in the internal-focus, external-focus, and control conditions. Importantly, no difference was observed between the internal-focus and external-focus conditions; however, both fixed focus conditions were associated with shorter swimming times than the control condition. In addition, attentional flexibility was higher in the switching condition than in the internal-focus, external-focus, and control conditions. Similarly, no difference was observed between the internal-focus and external-focus conditions; however, both fixed focus conditions were associated with higher flexibility than the no-instruction control condition.

With regard to swimming performance, the findings support Hypothesis 1 and extend previous attentional focus research by highlighting the functional importance of dynamic attentional regulation. Traditional attentional focus studies have frequently reported performance advantages for external over internal focus, often interpreted through the constrained action hypothesis, which posits that internal focus disrupts automatic motor control processes (McNevin et al., 2003; Wulf, 2013; Wulf and Lewthwaite, 2016). However, the present results challenge a strict internal–external dichotomy, as no performance difference was observed between internal-focus and external-focus conditions in beginner swimmers. We observed that the switching condition aligns with qualitative and theoretical accounts, suggesting that athletes naturally adjust attentional focus during task execution, particularly under challenging conditions (Nicklas et al., 2022). Previous qualitative studies have reported that performers switch between different attentional cues to maintain control and adapt to situational demands (Carson and Collins, 2016; Renshaw et al., 2010). The present findings extend this study by providing quantitative evidence that an explicitly instructed switching strategy enhances performance in novice swimmers. Given the continuous coordination demands and limited sensory feedback inherent in swimming, a switching focus strategy may allow beginners to attend to movement-related information when necessary while also directing attention toward task-level goals, thereby facilitating more efficient movement execution.

The results regarding attentional flexibility further clarify the mechanism underlying this performance advantage and support Hypothesis 2. Attentional flexibility was highest in the switching condition, whereas internal-focus and external-focus conditions did not differ from each other. This pattern suggests that attentional flexibility is not primarily driven by attentional content but by the ability to dynamically regulate attention (Lavie et al., 2004; Diamond, 2013). Fixed-focus strategies may inadvertently constrain attentional regulation by encouraging performers to adhere to a single attentional rule throughout the task. In contrast, switching focus instructions appear to promote a more adaptive attentional mode, enabling performers to adjust attentional allocation in response to ongoing task demands. We suggest that attentional flexibility may serve as a key mechanism linking attentional strategy to motor performance in beginner swimmers. Rather than interfering with automaticity, flexible attentional regulation may support coordination and error correction when stable motor patterns have not yet been fully established.

Importantly, the control analysis revealed no differences in anxiety levels across conditions. Anxiety has been shown to impair motor performance, particularly in novice performers (Masters, 1992; Beilock and Carr, 2001), and could plausibly explain performance differences if one condition reduced emotional arousal more effectively than others. However, the absence of condition effects on anxiety indicates that emotional factors are unlikely to account for the observed performance advantages. This finding strengthens the conclusion that switching focus improved performance through attentional rather than affective mechanisms. Thus, the switching strategy appears to enhance performance not by reducing swimmers’ anxiety but by enabling more effective regulation of attention during swimming.

From an applied perspective, the present findings have practical implications for coaching and instruction in early-stage swimming training. Coaches often prescribe specific attentional cues to improve technique or efficiency (Wulf, 2013; Wulf and Lewthwaite, 2016). The current results suggest that encouraging beginner swimmers to adopt a flexible attentional approach may be more beneficial than enforcing a single, fixed focus. Training programs that explicitly teach when and how to shift attention during performance may foster both attentional control and motor coordination. Such an approach may be particularly valuable in complex skills, such as swimming, where performers must continuously integrate sensory feedback, body position, and task demands.

Several limitations of the present study should be acknowledged. First, attentional flexibility was assessed using subjective ratings, which capture perceived rather than objective attentional dynamics. While the manipulation check indicated that participants completely adhered to the attentional-focus instructions based on self-reported measures, the dichotomous yes/no format did not capture the frequency, timing, or consistency with which participants applied or switched attentional focus during swimming. Therefore, future studies should use more detailed manipulation checks, such as trial-by-trial reports or post-trial verbal protocols, together with objective measures of attentional allocation, such as eye-tracking or neurophysiological indices, to document how switching-focus strategies are implemented during performance and to further elucidate the underlying mechanisms. Second, the study focused exclusively on beginner swimmers performing a short-distance task. It remains unclear whether the benefits of switching focus strategies generalize to longer distances, different swimming strokes, or more skilled performers. Future research should examine whether the role of attentional flexibility changes as motor skills become more automatized. In addition, future studies could investigate the effects of switching focus strategies on learning over time. The present study examined immediate performance effects, but repeated practice with a switching strategy may influence motor learning differently than fixed focus strategies. Longitudinal designs assessing both performance and learning outcomes would provide valuable insight into the durability and transferability of the attentional flexibility benefits.

In conclusion, the present study demonstrates that a switching focus strategy leads to superior swimming performance in beginner swimmers and that this advantage is closely associated with enhanced attentional flexibility rather than changes in anxiety. By emphasizing attentional flexibility as a key mechanism, these findings extend existing attentional focus theories beyond static internal–external distinctions and provide a more nuanced understanding of how attentional regulation supports motor performance during early skill development.

## Data Availability

The datasets presented in this article are not readily available because the study involved human participants, including minors, and individual-level data cannot be shared publicly due to ethical and privacy restrictions. Requests to access the data should be directed to the corresponding author.
